# Organisational caring ethical climate and its relationship with workplace bullying and post traumatic stress disorder: The role of type A/B behavioural patterns

**DOI:** 10.3389/fpsyg.2022.1042297

**Published:** 2022-11-02

**Authors:** Fang Jin, Ahsan Ali Ashraf, Sajid Mohy Ul Din, Umar Farooq, Kengcheng Zheng, Ghazala Shaukat

**Affiliations:** ^1^School of Management, Wuhan Polytechnic University, Wuhan, China; ^2^Department of Business Administration, University of Sialkot, Sialkot, Pakistan; ^3^Department of Business Education, University of Lahore, Lahore, Pakistan; ^4^Department of Business Education, University of Chenab, Gujrat, Pakistan; ^5^FAST School of Management, National University of Computer and Emerging Sciences, Faisalabad, Pakistan; ^6^School of Finance and Taxation, Zhongnan University of Economics and Law, Wuhan, China; ^7^Department of Sociology, University of Sindh, Jamshoro, Pakistan

**Keywords:** workplace bullying, caring climate, Type A personality, post traumatic stress disorder, PTSD, Type B personality

## Abstract

A multifaceted, holistic approach to identifying potential predictors is needed to eradicate workplace bullying. The current study investigated the impact of an unfavourable organisational climate that plays a role in breeding workplace bullying (social stressors). The present study also postulated that individual personality differences (Type A and Type B personality) mediate between a caring climate and workplace bullying. Similarly, the interaction between workplace bullying and personality impacts PTSD. We also checked the role of workplace bullying as a mediator between a caring climate and PTSD. This research tested all the proposed hypotheses (*N* = 298), and the study was conducted in Pakistan. The data is analysed using the two-step partial least square structural equation modelling (PLS-SEM) procedure. The first part assesses the measurement model, while in the second step, the structural model is evaluated. The results supported all the proposed hypotheses of this study. Type A behaviour moderated the caring climate—person-related bullying relationship, whereas it did not moderate the caring climate—work-related bullying in the suggested direction. Type A behaviour is moderated for both types of bullying and PTSD. Results also show significant indirect effects of a caring climate on PTSD through workplace bullying. This study will contribute theoretically to filling the literature gap on studies of climate-bullying and bullying-stress using contingency factors.

## Introduction

Workplace bullying is a critical and severe issue companies face today, impacting employees’ health and productivity. Alam and Abdin (2022) reported that US companies were losing approximately $300 billion per year due to the loss of employee productivity due to bullying. In contrast, it is as high as $600 billion for the UK. Similarly, 23% of employees in the UK, 25% in the US, and 37% in Australia witnessed workplace bullying (Vranjes et al., 2022b). Bullying is defined as the regular exploitation of a subordinate, co-worker, or senior in a hierarchy, which may lead to adverse social, psychological, and even physical consequences for the victims (Einarsen, 2000). Taris (2022) further argued that bullying is a time-dependent and contextual phenomenon. Initially, the preparator shows mild aggression that turns into hard-line aggression with time to become more explicit (Leymann, 1990; Vranjes et al., 2022a). Various studies explored the adverse effects of such regular exploitations from several perspectives, such as post-traumatic stress (Mikkelsen and Einarsen, 2002), anxiety (Rai and Agarwal, 2018), turnover intentions (Djurkovic et al., 2008), strain and wellbeing (Rai and Agarwal, 2018), depression and propensity to leave (Naseer et al., 2016), exhaustion and weak health (Hauge et al., 2007), isolation within group/teams (Samnani and Singh, 2012) and deviant work behaviour (Naseer et al., 2016).

To cope with its negative consequences, several studies also investigated the determinants of workplace bullying. These determinants range from individual-specific to team-related and organisational-level factors of bullying. This research postulates that organisational factors primarily act as a barrier or opportunity for bullying. Individual and team-related factors serve as catalysts for organisational factors to encourage or hamper bullying (Rai and Agarwal, 2018). In other words, individual and team-related factors moderate the relationship between organisational level determinants and workplace bullying.

Specifically, this research proposed that a caring climate (an organisational factor) is essential to bullying while the victim’s personality (an individual factor) moderates. A caring climate is defined as benevolence in terms of moral philosophy, where employees have a sincere concern for the wellbeing of others (Martin and Cullen, 2006). Wimbush and Shepard (1994) contended that a caring climate is related to employees’ ethical, dysfunctional, and counterproductive behaviours. A caring climate may impose ethical, moral, and organisational restrictions on bullies not to harm others and to create an environment of respect, trust, and wellbeing (Wimbush and Shepard, 1994). Therefore, the existence or absence of such a caring climate could control or encourage bullying behaviours. However, the probability of bullying in the existence or absence of a caring climate may depend on the target’s personality.

For instance, Type A (a personality trait) individuals are outgoing, determined, aggressive, impatient, rigidly organised, competitive, and workaholics (Friedman and Rosenman, 1974). If Type A individuals fail to achieve their objectives, then the hard-driven and go-getter nature of their Type A personality can make them more vulnerable to developing perceptions of being bullied, even in a caring climate. Conversely, Type B individuals remain relaxed and untroubled in a politicised environment and work pressures (Kamdar and Van Dyne, 2007). Therefore, their patience and less aggressive and friendly behaviour can resist the bullying attacks even without a caring climate. If these notions are true, it can be theorised that the target personality moderates the relationship between a caring climate and workplace bullying.

Similarly, personality can also play a vital role in controlling the negative consequences of workplace bullying. Since Type A individuals are more impatient and workaholics, these individuals experience more stress when bullied. Conversely, Type B remained relaxed and carefree in response to bullying, which affected them less from a stress perspective. Thus, it is also proposed that target personality also moderates the relationship between workplace bullying and stress.

The purpose of this research is to investigate the aforementioned moderating effects in the case of Pakistan. In developing countries, stringent HR policies have not been developed to control workplace bullying. Einarsen et al. (2002) argued that developing countries continuously face challenges in the situational dynamics of society. Such a harsh reality applies to workplace bullying in developing countries like Pakistan. Therefore, studying the consequences and determinants of workplace bullying is a much-needed research agenda, especially in developing countries. However, various studies have addressed this issue but ignored the contingency factors while studying the consequences and determinants of workplace bullying. Notably, in their meta-analysis, Rai and Agarwal (2018) explored that personality is one of the most important but less researched contingency factors in this respect.

## Theoretical background

In the last few years, bullying in the workplace has emerged as a field of research distinct from sexual and racial harassment (Einarsen et al., 2009). Workplace bullying is defined as the regular exploitation of a subordinate, co-worker, or someone senior in a hierarchy, which may lead to adverse social, psychological, and even physical consequences for the victims (Einarsen, 2000). This definition of bullying consists of three aspects. The first part, ‘regular exploitation’, highlights bullying as a time-dependent phenomenon. Here, ‘exploitation’ can be defined as interpersonal mistreatment (Einarsen et al., 2011), while ‘regular’ refers to the frequency of its occurrence. Therefore, according to the definition, exploitation (i.e. interpersonal mistreatment) will become workplace bullying when it occurs regularly (i.e. regularly, such as weekly, and over some period of time, such as six months).

The second part of the definition elaborates on the victim or the perpetrator as a subordinate, co-worker, or senior (Evans, 1985, Einarsen, 1999; Sullivan, 2012). In other words, workplace bullying can occur from supervisor to subordinate (Einarsen, 2000), from subordinate to supervisor (Mathisen et al., 2011), and between co-workers (Fox and Stallworth, 2005). The third part of the definition delineates the negative consequences of bullying. For instance, targets of bullying are more likely to have Post-Traumatic Stress Disorder (PTSD). It is a severe anxiety disorder associated with persistent exposure to stressful conditions. PTSD is characterised by a triad of symptoms, i.e. hyper-arousal, re-experiencing stressful events through nightmares, avoidance, and denial (Kerasiotis and Motta, 2004). Studies show that, on average, 86% of bullying victims report symptoms, such as memory problems, nervousness, social isolation, avoidance, and PTSD (Leymann, 1990; Mikkelsen and Einarsen, 2002).

Since bullying can have severe negative consequences, various studies have explored firm-specific, team-level, and organisational-level determinants of workplace bullying to develop mitigating strategies. Individual-specific factors include both target-specific and perpetrator-specific factors. For instance, Persson et al. (2009) have found that neurotic and extroverted individuals are more susceptible to bullying. Similarly, employees (as the perpetrators) with high-stress jobs, high workloads, and low job autonomy are more likely to engage in bullying behaviours (Baillien et al., 2011). Literature has also highlighted group-related risk factors, such as group norms, status inconsistency, and certain situational factors (e.g., task conflict) as determinants of bullying (Samnani and Singh, 2012). Employees within the group are adversely affected because of their close exposure to such behaviours as witnesses (Einarsen et al., 1994; Lutgen-Sandvik et al., 2007). Similarly, organisational level risk factors such as leadership styles, organisational culture, ethical climate, organisational policies, and situational factors have been studied as antecedents of bullying (Rai and Agarwal, 2018).

This research postulates that organisational-level factors are the main determinants of workplace bullying. Mainly, organisational culture and climate provide workplace bullying barriers or opportunities. Einarsen (2000) claimed that the probability of bullying would be significantly lower if the organisation’s culture did not encourage or support such behaviours. If there were strict penalties for such behaviour and both victims and bullies knew about them, the chances of bullying would be lower. Similarly, bullying may be considered acceptable in some organisational cultures, or a wrong interpretation of organisational culture may result in a wrong understanding of bullying and expected behaviour. In other words, acceptability, tolerance, and interpretation of behaviour may depend on organisational culture (Hauge et al., 2007). The same arguments can be valid for the organisational climate, which is more concrete and explicit than the organisational culture.

Wimbush and Shepard (1994) found a significant relationship between bullying and organisational climate. They contended that an ethical climate is related to ethical, dysfunctional, and counterproductive behaviours. Employees within a caring climate (a dimension of organisational climate) are not likely to behave detrimentally to others, as the underlying assumption of a caring climate is benevolence in terms of moral philosophy and utilitarianism (Wimbush and Shepard, 1994; Cullen et al., 2003; Martin and Cullen, 2006). Since employees are more interested in others’ wellbeing and less likely to engage in harmful behaviour, the chances of workplace bullying are also low within a caring climate.

However, Rai and Agarwal (2016) argued that studying the consequences and determinants of workplace bullying with linear effects is inappropriate. Rai and Agarwal (2016) argued that workplace bullying is considered a misunderstood and oversimplified concept. They further contended that studying linear relationships between workplace bullying and its determinants and consequences is a prime reason for misunderstandings and oversimplifications of workplace bullying. Since behaviours do not change linearly, some contingency factors (such as precise psychological mechanisms) can affect the interpretations of bullying, its determinants, and its consequences. Hence, studying workplace bullying with intervening mechanisms will provide a deep understanding of the construct within a specific context.

Samnani and Singh (2012) revealed that the literature on determinants and consequences of bullying without mediating and moderating factors is rich. However, few studies used mediating and significantly moderating factors in studying both the determinants and consequences of workplace bullying. For instance, Rai and Agarwal (2016) explored 53 studies using mediating or moderating factors in this context. Among these, 14 studies investigated some moderating variable for bullying-outcomes relationships, while only nine articles explored the antecedents-bullying relationship using some moderator. These statistics show that few studies explain the outcomes and determinants of bullying using intervening mechanisms. The systematic review of Rai and Agarwal (2016) also found that no study had investigated the climate-bullying relationship using contingency factors.

This research intends to fill this gap by studying climate-bullying and bullying-stress relations using contingency factors. We propose a theory of determinants of workplace bullying with an intervening mechanism. It is postulated that organisational level variables provide an opportunity or barrier for workplace bullying, while individual-specific and team-related factors increase or decrease its intensity. We tested this theory for organisational climate (an organisational level variable) and target personality (an individual-specific variable). This research proposes that organisational climate provides the opportunity/barrier for bullying while the victim’s personality affects the intensity of such an opportunity or barrier. The determined and aggressive personalities of Type A individuals make themselves more vulnerable to frustration and provocation if they do not achieve their objectives (Byrne, 1996). Therefore, Type A individuals may view normal behaviour as bullying, even in a caring climate where employees care for others’ wellbeing and barriers are levied on harmful behaviours. Similarly, in the absence of a caring climate, Type A individuals can be more vulnerable to bullying due to aggressive, frustrated, and intolerant behaviour.

Conversely, personality B individuals may act the opposite and resist bullying even without a caring climate because these individuals lack characteristics, such as impatience, aggression, and hostility. If these postulations are true, the target personality can be explained as a moderator for climate-bullying relations. The first objective of this research is to test this relationship using the following hypothesis.

**H1:** Target personality moderates the relationship between a caring climate and both types of Bullying.

We also tested the moderating role of personality in the bullying-stress relationship. It is argued that Type A individuals are more susceptible to the negative consequences of bullying. It is because Type A individuals tend to stress out if they continuously think they can be bullied, compared to Type B individuals who would rather stay relaxed and ignore such adverse behaviour. The second objective of this research is to investigate the moderating role of personality in bullying-stress relations using the following hypothesis.

**H2:** Target personality moderates the relationship between both types of bullying and Post-Traumatic Stress Disorder (PTSD).

Here, it is also notable that if a caring climate affects bullying and bullying affects stress, bullying might be seen as a mediator between climate and stress. The study of this mediating effect will help to understand the role of organisational climate as a root cause of stress. The third objective of this research is to test the mediating role of bullying between a caring climate and stress using the following hypothesis.

**H3:** Bullying mediates the relationship between a caring climate and Post-Traumatic Stress Disorder (PTSD).

**FIGURE 1:**
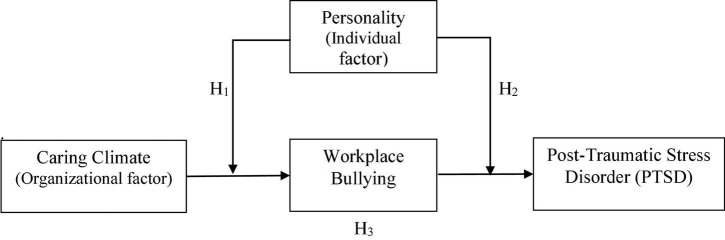
Theoretical framework.

Figure 1 represents the theoretical framework and hypotheses of the proposed study. These three objectives’ results can help policymakers control bullying and stress by creating a caring climate in the contingency of individual personalities within an organisation.

## Methodology

### Participants

The data were taken from public and private organisations in Lahore, Pakistan. Non-probability, more precisely convenient, Sampling technique was used to collect the data. The organisations that participated in this study include public and private hospitals, universities, banks, and pharmaceutical organisations. A letter describing the purpose and objective of the study was attached with the survey that assured the anonymity and confidentiality of respondents as well. All respondents participated in this research voluntarily. Out of 500 questionnaires distributed, 298 completed usable were given back, yielding a response rate of 60%. Table 1 shows the characteristics of the respondents in terms of gender, age, and industry. The results show that 59.7% of the respondents for this study were women, and most of the respondents were 26–35 years old.

**TABLE 1:** Descriptive statistics.

Variable	Category	Frequency	Percent	Cumulative percent
Gender	*Female*	178	59.7	59.7
	*Male*	120	40.3	100.0
Age	*Below 25*	75	25.2	25.2
	*26-35*	144	48.3	73.5
	*36-45*	66	22.1	95.6
	*46-55*	10	3.4	99.0
	*Above 65*	3	1	100.0
Industry	*Banks/Manufacturing*	75	25.2	25.2
	*Education*	63	21.1	46.3
	*Medical*	160	53.7	100.0

Moreover, 53.7% of respondents were from the medical profession. Bartlett et al. (2001) argued that the sample size ranges from a minimum of two hundred for simple relationships and a complex model; a 400 sample size is appropriate (Boomsma, 1983). Moreover, we also deploy the G* Power sampling technique, and according to the great power of 0.05 and a medium-size effect of 0.15, the minimum sample size should be 200 according to the predictors of the study. However, we were able to approach more respondents who were 298, which is greater than the minimum requirement (Kettling et al., 2014).

### Material

The data were collected through a self-administrated questionnaire. As a precautionary measure and to get a good response from the respondents, the author personally visited the respondents with the questionnaire to explain and respond to any ambiguity in questions. The questionnaire is divided into two main sections. The first part included questions regarding personal data, while the second part asked questions regarding workplace bullying, a caring climate, personality, and stress. The details of these questions asked for each construct are given below.

#### Workplace bullying

Workplace bullying is measured using the shortened version of the Negative Acts Questionnaire, consisting of twenty-two items (Einarsen et al., 2009). Respondents were asked how often they experienced hostile acts at work over the past six months. Sample items included “Someone withholding information which affects your performance,” “Being ignored or facing a hostile reaction when you approach,” and “Having allegations made against you.” The response scale had anchors ranging from 1 (Never) to 5 (Daily). Following Gupta et al. (2017), a 22-item questionnaire is further segregated into three dimensions of workplace bullying, including workplace bullying (8 items), personal bullying (11 items), and physical bullying (3 items). Workplace bullying refers to abusing others through unjustified tasks, while personal and physical bullying is more severe and targets the victim directly.

#### Caring climate

The Ethical Climate Questionnaire developed by Victor and Cullen (1988) measures a caring climate. On a five-point Likert-type scale, responses ranged from *strongly agree* to *strongly disagree*. Sample items are *what is best for everyone in the company is a major consideration here*; *In the company, each person is expected above all to work efficiently*; “In this company, people protect their interests above all else; *work is considered substandard only when it hurts the company’s interests*, and response choices ranged from 1 (Strongly Disagree) to 5 (Strongly Agree).

#### Post-traumatic stress disorder

We did not focus on traditional job stress or anxiety measures to measure stress. Instead, we focus on post-stress disorder due to bullying. Therefore, a 22-item self-reported questionnaire, “the Impact of Event Scale-Revised (IES-R)” by Rosner and Hagl (2008), is used to measure stress. This questionnaire is a revised version of the original Horowitz (IES) to better capture the DSM-IV criteria for PTSD. Sample items included *Any reminder that brought that back feeling about it*; *I had trouble falling asleep*; *I had dreams about it*; and response anchors were from 1 “Not at all” to 5 “Extremely.”

#### Target personality

The big five models of personality have been used intensively in the literature on personal behaviours (Goldberg, 1990; Costa and McCrae, 1992). However, James and LeBreton (2010) and Bruck and Allen (2003) are among the opponents of the big five model. They argued that the big five model does not account for individual differences, motives, and interests. Bruck and Allen (2003) further argued that Type A & Type B behavioural patterns are more helpful in explaining individual behaviours. It is because Type A behavioural pattern explains the combination of different personality traits rather than individual traits. Therefore, this research followed their approach and measured target personality using a twelve-item scale developed by Spence et al. (1987). Sample items included *I consider myself hard-driving*; *How much does the workplace stir you into action?*; and *compared with other students, I approach life in general*. Response scale anchors ranged from 1 D (Strongly Disagree) to 5 D (Strongly Agree).

### Procedure

The data is analysed using a two-step partial least square structural equation modelling (PLS-SEM) procedure. The first part assesses the measurement model, while in the second step, the structural model is evaluated. PLS-SEM is used due to its soft distributional assumption and ability to analyse complex relationships simultaneously in a standalone model. To assess the significance of the measurement model and the proposed relationships, we applied a bootstrapping procedure of 5000 subsample simulations. To perform and report the impactful analysis, we followed the recommendations of Benitez et al. (2019).

## Results and discussion

### Measurement model

The measurement model is analysed from four perspectives, i.e., indicators’ reliability, discriminant validity, construct reliability, and convergent validity. Indicator reliability is analysed using outer loadings and outer weights. Table 2 shows that all the included items show factor loadings of 0.6 or above. We excluded all the items that showed outer loadings of less than 0.6. For instance, there were twelve items for the personality, but we selected only four items showing factor loadings of more than 0.6 in the final measurement model. Similarly, cross-loadings are analysed to confirm the discriminant validity of all the constructs. Results revealed that all the items of a caring climate (CC), personality (Pers), personal bullying (PB), workplace bullying (WB), and PTSD showed low outer loadings across the constructs. Notably, Table 2 shows the results for only two dimensions of bullying, i.e., workplace bullying and personal bullying, while no data are provided for physical bullying. The initial analysis showed that all items of physical bullying and some items of personal bullying and workplace bullying exhibited high cross-loadings. Such items are excluded, and only six items of personal bullying and three items of workplace bullying showing high outer loadings (greater than 0.6) and low cross-loadings are included in the final model.

**TABLE 2:** Cross loading and Heterotrait-Monotrait Ratio (HTMT).

	Cross loading						HTMT			
	Items	CC	PB	Pers	Stress	WB	CC	PB	Pers	PTSD
CC	Q5.5	**0.819**	0.423	0.355	–0.324	0.323				
	Q5.6	**0.772**	0.384	0.353	–0.202	0.263				
	Q5.1	**0.766**	0.375	0.260	–0.040	0.207				
	Q5.2	**0.754**	0.358	0.258	–0.131	0.197				
	Q5.3	**0.732**	0.287	0.236	–0.145	0.274				
	Q5.4	**0.686**	0.271	0.279	–0.164	0.243				
	Q5.7	**0.679**	0.366	0.128	–0.053	0.269				
PB	Q3.12	0.382	**0.828**	0.263	–0.280	0.350	0.541			
	Q3.11	0.437	**0.824**	0.296	–0.314	0.339				
	Q3.13	0.400	**0.802**	0.224	–0.347	0.305				
	Q3.5	0.311	**0.733**	0.166	–0.138	0.412				
	Q3.7	0.345	**0.732**	0.128	–0.146	0.378				
	Q3.6	0.257	**0.6**	0.102	–0.085	0.255				
Pers	Q6.9	0.289	0.196	**0.832**	–0.419	0.332	0.443	0.324		
	Q6.11	0.306	0.258	**0.798**	–0.379	0.221				
	Q6.10	0.288	0.192	**0.794**	–0.321	0.267				
	Q6.8	0.227	0.183	**0.616**	–0.316	0.325				
PTSD	Q4.15	–0.199	–0.282	–0.345	**0.809**	–0.237	0.237	0.320	0.542	
	Q4.18	–0.171	–0.276	–0.373	**0.798**	–0.199				
	Q4.16	–0.134	–0.276	–0.369	**0.787**	–0.309				
	Q4.2	–0.117	–0.164	–0.310	**0.766**	–0.174				
	Q4.20	–0.236	–0.306	–0.422	**0.762**	–0.252				
	Q4.3	–0.078	–0.106	–0.220	**0.748**	–0.173				
	Q4.14	–0.153	–0.243	–0.277	**0.747**	–0.166				
	Q4.19	–0.195	–0.278	–0.432	**0.746**	–0.257				
	Q4.9	–0.202	–0.191	–0.398	**0.734**	–0.291				
	Q4.4	–0.143	–0.161	–0.235	**0.731**	–0.218				
	Q4.17	–0.075	–0.173	–0.361	**0.729**	–0.233				
	Q4.21	–0.243	–0.329	–0.501	**0.724**	–0.367				
	Q4.1	–0.011	–0.084	–0.264	**0.717**	–0.165				
	Q4.22	–0.138	–0.161	–0.385	**0.713**	–0.177				
	Q4.5	–0.187	–0.206	–0.327	**0.711**	–0.244				
	Q4.13	–0.129	–0.214	–0.266	**0.700**	–0.090				
	Q4.10	–0.202	–0.264	–0.368	**0.698**	–0.343				
	Q4.6	–0.147	–0.201	–0.333	**0.694**	–0.213				
	Q4.12	–0.191	–0.259	–0.308	**0.650**	–0.112				
	Q4.7	–0.102	–0.193	–0.264	**0.624**	–0.220				
WB	Q3.2	0.256	0.366	0.326	–0.263	**0.874**	0.404	0.539	0.480	0.341
	Q3.3	0.329	0.400	0.315	–0.341	**0.862**				
	Q3.1	0.293	0.373	0.325	–0.188	**0.821**				

This table provides the results for discriminant validity criteria of cross loading and Heterotrait-Monotrait Ratio (HTMT). Here, CC, PB, Pers, and WB refer to a caring climate, personal bullying, personality, and workplace bullying, respectively. PTSD shows post-traumatic stress disorder. The bold values show a strong correlation with the same construct and weak correlation with different construct indicating discriminant validity.

Similarly, the Heterotrait-Monotrait Ratio (HTMT) is used to analyse the discriminant validity of the measurement model. It is an evaluation of factor association (on upper boundary) correlation. HTMT is used to understand the differences between two factors. Its value should be smaller than 1 to ensure discriminant validity. The results of the HTMT ratio are provided in Table 2. The results showed that all the values of HTMT are less than 1, indicating acceptable discriminant validity. Hence, the measurement model fulfils indicator reliability and discriminant validity criteria. To assess the internal consistency of the measurement model, we used Cronbach’s alpha (CA) and composite reliability (CR). Table 3 shows that the values of CA and CR of all the latent variables are above 0.76 and 0.85, respectively. Since these values are more significant than the minimum threshold of 0.7, it can be concluded that the data is internally reliable. Convergent validity is assessed through average variance extracted (AVE). Results show that the AVE values for all the latent variables are more significant than the benchmark criteria of 0.5. Hence, it can be inferred that the measurement model also fulfils the convergent validity criteria.

**TABLE 3:** Reliability and VIF.

	Reliability and convergent validity			VIF		
	CA	CR	AVE	PB	WB	PTSD
CC	0.87	0.90	0.56	1.29	1.15	1.56
WB	0.81	0.89	0.73	1.24		1.44
PB	0.85	0.89	0.57			1.62
Personality	0.76	0.85	0.58	1.27	1.15	1.31
PTSD	0.95	0.96	0.53			

Table 3 also shows that the inner VIFs of all the variables are less than three, showing the appropriateness of the measurement model for structural modelling.

### Structural model

**FIGURE 2:**
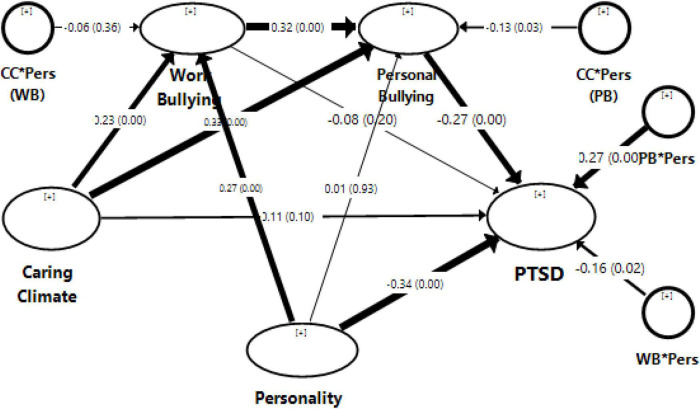
Structural model. The above figure is a structural model exported from SmartPLS. In the figure, circles represent the moderating variables. The strength of the arrows shows the relative importance of the relationships. Path coefficients along with significance in parentheses are also provided at each arrow.

Figure 2 is the graphical representation of the structural model with path coefficients and their significance values. The detailed results of the structural model are presented in Table 4. The validity of structural models is assessed through R2 and f2. Table 4 shows that the R2 of the model using stress as a dependent variable is 0.34 or 34%. This shows that 34% of the variations in stress are due to the proposed model. Results show that the direct effect of a caring climate (CC) on stress (PTSD) is insignificant. However, the relationship between a caring climate and both types of bullying is significantly positive. Here it is notable that high scores of CC represent more application of a caring climate, while high scores of bullying show a low probability of bullying. Therefore, the positive CC-bullying relationship can be interpreted as a ‘low chance of bullying for organisations having a more caring climate and vice versa. Employees within a caring climate show benevolent behaviour regarding moral philosophy and utilitarianism. Consequently, employees working in a caring climate do not get involved in bullying activities. However, the target personality can change this postulation.

**TABLE 4:** Structural model statistics.

	Path coefficient	T statistics	*P* values	f2
CC - > PTSD	0.107	1.670	0.095	0.011
CC - > PB	0.328	6.155	0.000	0.125
CC - > WB	0.225	4.001	0.000	0.053
CC*Pers - > PB	–0.125	2.158	0.031	0.023
CC*Pers - > WB	–0.063	0.904	0.366	0.005
PB - > PTSD	–0.272	4.514	0.000	0.070
WB - > PTSD	–0.079	1.293	0.196	0.007
PB*Pers - > PTSD	0.272	6.408	0.000	0.104
WB*Pers - > PTSD	–0.156	2.264	0.024	0.034
Pers - > PB	0.006	0.089	0.929	0.000
Pers - > WB	0.274	4.655	0.000	0.077
Pers - > PTSD	–0.342	6.246	0.000	0.138
WB - > PB	0.316	6.013	0.000	0.121

	**Adj R square**			

PB	0.324			
PTSD	0.34			
WB	0.186			

**FIGURE 3:**
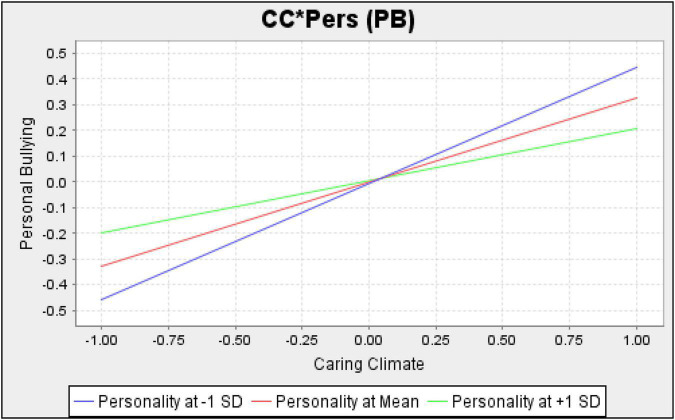
Personality as a moderator between a caring climate and personal bullying.

Table 4 shows that the cross-product of a caring climate and personality (CC*Pers - > PB) is significantly harmful to personal bullying. Her personality is divided into two types: Type A and Type B. A high-score personality represents an inclination to Type B, while low scores characterise a Type A personality. Figure 3 depicts this moderation effect graphically using three values of personality. Figure 3 shows that the regression line for Type B (at + 1 SD) is higher than that of Type A (at −1 SD) for low scores of a caring climate. These results conclude that personal bullying is expected more frequently for Type A than for Type B in the absence of a caring climate. However, the regression line for Type B (at + 1 SD) is lower than that for Type A (at −1 SD) for high scores of a caring climate. Thus, in a highly caring climate, personal bullying is expected to occur less frequently for Type A than for Type B. These results infer that personality moderates the relationship between a caring climate and personal bullying. The reason for this moderation can be Type A’s aggressive behaviour and Type B’s calmness. Due to their determined and aggressive personalities, Type A may view normal behaviour as bullying without a caring climate. Similarly, Type B’s calm nature may ignore bullying without a caring climate. As a result, personal bullying can be expected more frequently for Type A personalities than Type B in the absence of a caring climate.

However, Type A’s competitive and workaholic nature may help them complete their tasks in a strong, caring climate. Consequently, it is less expected that Type A perceives normal behaviour as personal bullying within a supportive, caring climate. Hence, we accept our first hypothesis that personality moderates a caring climate and personal bullying. Table 4 also shows that the personality moderating effect is insignificant for workplace bullying (CC*Pers - > PB). Since Type A individuals are rigidly organised, competitive, and workaholics, they might not view work-related things as bullying. They might like to do more work and respond aggressively when their tasks are not completed. Consequently, their perception is changed only by personal bullying. Thus, we reject the personality hypothesis as a moderator for the relationship between a caring climate and workplace bullying.

**FIGURE 4:**
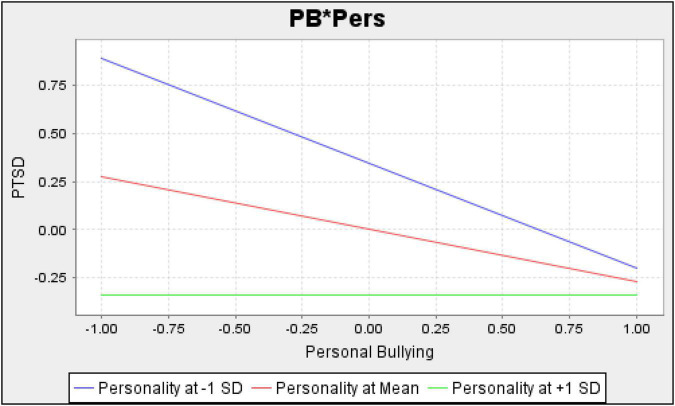
Personality as a moderator between personal bullying and post traumatic stress disorder.

**FIGURE 5:**
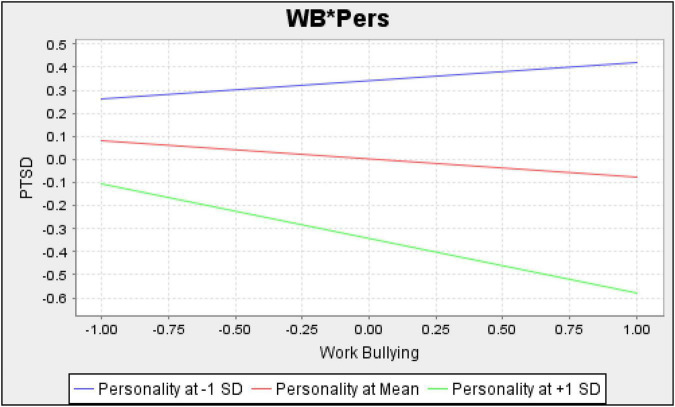
Personality as a moderator between personal bullying and post traumatic stress disorder.

Table 4 further explores that personal bullying (PB) and workplace bullying (WB) have significantly negative and insignificant relation to stress (PTSD), respectively. This indicates that employees suffer PTSD when the chances and frequency of personal bullying increase. However, the intensity of such negative consequences of personal and workplace bullying depends on the target personality. Table 4 shows that personality significantly moderates the relationship between bullying and PTSD. Figures 4, 5 provide graphical representations of such moderating effects when personality scores are mean, + 1 SD, and −1 SD.

Figure 4 depicts a straight regression line for personal bullying and PTSD when personality is at + 1 SD from its mean. However, for Type A (at −1 SD), the regression line for personal bullying and PTSD is more downward. These results reveal that frequent personal bullying leads to more stress for Type A individuals. In contrast, personal bullying has no effect on stress for Type B. Aggressive and impatient behaviour of Type A may make them more stressed in response to personal bullying. Conversely, Type B’s calm behaviour absorbs personal bullying’s negative effects. Hence, we accept our second personality hypothesis as a moderator between personal bullying and stress.

Figure 5 provides the results of workplace bullying and PTSD while considering the contingency of personality. Figure 5 shows a slight upward slope for personality at −1 SD. This shows that frequent workplace bullying decreases PTSD for Type A. It is possible that workplace bullying provides opportunities for Type A personalities to show their competitive and workaholic nature. Thus, after completing tasks in workplace bullying, Type A may feel more satisfaction and less stress.

Conversely, Figure 5 depicts a downward regression line for Type B (at + 1 SD). This shows that frequent workplace bullying increases PTSD for Type B. Since workplace bullying affects PTSD with varying intensities at different personality levels, we also accept our second hypothesis of personality as a moderator between workplace bullying and stress. These results also support our general theory that organisational factors are the primary source of bullying while individual-specific factors act as moderators. However, future studies are recommended to test this theory for other organisational and individual-specific factors.

This research’s third hypothesis is to test the mediating role of bullying between a caring climate and PTSD. Table 4 concludes the insignificant direct effect of the caring climate on PTSD. A caring climate may affect personal and workplace bullying and PTSD. Such indirect effects are presented in Table 5. The results show a significant indirect effect of a caring climate through personal bullying only. However, the indirect effect of a caring climate on workplace bullying is insignificant. Thus, we accept our third hypothesis of personal bullying as a mediator between a caring climate and PTSD.

**TABLE 5:** Specific indirect effects.

	Indirect effect	T statistics	*P* values
CC - > PB - > PTSD	−0.089	3.330	0.001
CC - > WB - > PTSD	−0.018	1.224	0.221
CC - > WB - > PB - > PTSD	−0.019	2.556	0.011

## Conclusion

This research proposes a new way of understanding the determinants and consequences of workplace bullying. The organisational’s caring climate is a primary source for directly or indirectly creating or stopping the unfavourable social stressor named workplace bullying. In contrast, individual factors can moderate the relationship between determinants and consequences of workplace bullying. We selected a caring climate as an organisational level determinant and post-traumatic stress disorder as a consequence of workplace bullying in the contingency of the target personality. We tested our proposed model by using a self-administrative questionnaire, and we used PLS-SEM for data analysis. Results are consistent with our proposed model in that a caring climate did not directly impact PTSD, but indirect relationships were seen through workplace bullying. Another finding shows that the role of a Type A personality as a moderator between a caring climate and workplace bullying is significant. That represents the notion that Type A individuals influence the relationship between a caring climate and workplace bullying. Finally, the cross effect of Type A personality with workplace bullying impacts PTSD.

## The implication of the study

This research is significant from both theoretical and practical perspectives. The investigation of personality (an individual factor) as a moderator between a caring climate (an organisational level factor) and bullying will provide a theoretical contribution to a less researched area. Our results also support our theory that individual factors moderate organisational-level factors and workplace bullying. This theoretical underpinning also invites the researchers to test our proposed theory for other individuals, team-related and organisational factors in the context of workplace bullying. Conversely, a sample from Pakistan will provide practical implications regarding HR policies in developing countries. This research will guide practitioners in controlling workplace bullying by creating an appropriate organisational climate while considering the individual personality. Controlling bullying behaviours will increase individual and organisational performance, ultimately bringing social and economic prosperity to developing countries.

## Data availability statement

The raw data supporting the conclusions of this article will be made available by the authors, without undue reservation.

## Ethics statement

The studies involving human participants were reviewed and approved by Kaleem Ahmed and Zeeshan Ahmed. The patients/participants provided their written informed consent to participate in this study.

## Author contributions

FJ: introduction. AAA: idea creation and literature review. SMU: analysis and interpretation. UF: discussion. KZ: supervision and conclusion. GS: proof-reading. All authors contributed to the article and approved the submitted version.
