# Corrigendum: Organisational caring ethical climate and its relationship with workplace bullying and post traumatic stress disorder: The role of type A/B behavioural patterns

**DOI:** 10.3389/fpsyg.2023.1137029

**Published:** 2023-01-25

**Authors:** Fang Jin, Ahsan Ali Ashraf, Sajid Mohy Ul Din, Umar Farooq, Kengcheng Zheng, Ghazala Shaukat

**Affiliations:** ^1^School of Management, Wuhan Polytechnic University, Wuhan, China; ^2^Department of Business Administration, University of Sialkot, Sialkot, Pakistan; ^3^Department of Business Education, University of Lahore, Lahore, Pakistan; ^4^Department of Business Education, University of Chenab, Gujrat, Pakistan; ^5^FAST School of Management, National University of Computer and Emerging Sciences, Faisalabad, Pakistan; ^6^School of Finance and Taxation, Zhongnan University of Economics and Law, Wuhan, China; ^7^Department of Sociology, University of Sindh, Jamshoro, Pakistan

**Keywords:** workplace bullying, caring climate, Type A personality, post traumatic stress disorder, PTSD, Type B personality

In the published article, there was an error in affiliation 5 as published. Instead of “School of Management, FAST School of Management, Lahore, Pakistan,” it should be “FAST School of Management, National University of Computer and Emerging Sciences, Faisalabad, Pakistan.”

The authors apologize for this error and state that this does not change the scientific conclusions of the article in any way. The original article has been updated.

